# Results of a Saxitoxin Proficiency Test Including Characterization of Reference Material and Stability Studies

**DOI:** 10.3390/toxins7124852

**Published:** 2015-11-25

**Authors:** Kirsi Harju, Marja-Leena Rapinoja, Marc-André Avondet, Werner Arnold, Martin Schär, Werner Luginbühl, Anke Kremp, Sanna Suikkanen, Harri Kankaanpää, Stephen Burrell, Martin Söderström, Paula Vanninen

**Affiliations:** 1 VERIFIN (Finnish Institute for Verification of the Chemical Weapons Convention), Department of Chemistry, P.O. Box 55, A. I. Virtasen aukio 1, University of Helsinki, FI-00014 Helsinki, Finland; marja-leena.rapinoja@helsinki.fi (M.-L.R.); martin.soderstrom@helsinki.fi (M.S.); paula.vanninen@helsinki.fi (P.V.); 2 Federal Department of Defence, Civil Protection and Sport, SPIEZ LABORATORY, Austrasse 1, CH-3700 Spiez, Switzerland; marc-andre.avondet@babs.admin.ch (M.-A.A.); werner.arnold@babs.admin.ch (W.A.); martin.schaer@babs.admin.ch (M.S.); 3 ChemStat, Aarstrasse 98, CH-3005 Bern, Switzerland; info@chemstat.ch; 4 Finnish Environment Institute, Marine Research Centre, Erik Palménin aukio 1, FI-00560 Helsinki, Finland; anke.kremp@ymparisto.fi (A.K.); sanna.suikkanen@ymparisto.fi (S.S.); 5 Finnish Environment Institute, Marine Research Centre, Hakuninmaantie 6, FI-00430 Helsinki, Finland; harri.kankaanpaa@ymparisto.fi; 6 Marine Institute, Marine Environment and Food Safety Services, Rinville, Oranmore, County Galway, Ireland; stephen.burrell@marine.ie

**Keywords:** paralytic shellfish poisoning toxins, saxitoxin, proficiency test, dinoflagellate, mussel

## Abstract

A saxitoxin (STX) proficiency test (PT) was organized as part of the Establishment of Quality Assurance for the Detection of Biological Toxins of Potential Bioterrorism Risk (EQuATox) project. The aim of this PT was to provide an evaluation of existing methods and the European laboratories’ capabilities for the analysis of STX and some of its analogues in real samples. Homogenized mussel material and algal cell materials containing paralytic shellfish poisoning (PSP) toxins were produced as reference sample matrices. The reference material was characterized using various analytical methods. Acidified algal extract samples at two concentration levels were prepared from a bulk culture of PSP toxins producing dinoflagellate *Alexandrium ostenfeldii*. The homogeneity and stability of the prepared PT samples were studied and found to be fit-for-purpose. Thereafter, eight STX PT samples were sent to ten participating laboratories from eight countries. The PT offered the participating laboratories the possibility to assess their performance regarding the qualitative and quantitative detection of PSP toxins. Various techniques such as official Association of Official Analytical Chemists (AOAC) methods, immunoassays, and liquid chromatography-mass spectrometry were used for sample analyses.

## 1. Introduction

Establishment of Quality Assurance for the Detection of Biological Toxins of Potential Bioterrorism Risk (EQuATox) was a 36 month project (1 January 2012–31 December 2014) under the 7th European Union Framework Programme for Research (FP7) coordinated by the Robert Koch-Institut (Berlin, Germany). The project consisted of four separate proficiency tests (PTs) on four different toxin types: ricin, saxitoxin (STX), staphylococcal enterotoxin B (SEB), and botulinum neurotoxin (BoNT). The aim of the EQuATox project was to develop methods, procedures, and protocols for the analysis of selected chemical and biological substances allowing a comparison of results from different laboratories. The STX PT was organized by the Finnish Institute for Verification of the Chemical Weapons Convention, University of Helsinki, Finland (VERIFIN). STX is a highly poisonous non-peptide, low molecular weight toxin at the interface of classical biological and chemical agents; it is a well-defined substance under both the Chemical Weapons Convention (CWC, Schedule 1) and the Biological Weapons Convention (BWC). It is produced by some species of cyanobacteria and marine dinoflagellates e.g., *Alexandrium ostenfeldii* in the Baltic Sea [1]. STX accumulates in seafood, and it may also contaminate drinking water, thus causing severe health problems. Saxitoxin is a neurotoxin which blocks sodium channels, and intoxication may lead to paralysis and even death. Numerous fatal cases of paralytic shellfish poisoning (PSP) toxins have been reported globally [2], but the improved monitoring of microalgae and PSP toxins in shellfish has decreased the risks. Saxitoxin has also been considered to be a potential bioterrorism risk [3,4]. At least 57 structurally different analogues of STX are listed in a recent literature review [5]. The commercial availability of certified reference material is limited only to STX and about 10 of its analogues, which are most commonly produced by microalgae and most likely present in seafood. Toxicity equivalent factors have been applied to calculate detected analogues as STX equivalents for monitoring purposes using HPLC with fluorescence detection (FLD), though the acute toxicity of STX and its analogues via oral administration do not always correlate with the mouse bioassay (MBA) results, and e.g., the toxicity of neosaxitoxin (NEO) by i.p. injection seems to be higher than calculated from the toxicity equivalent factors [6]. The regulatory limit (800 µg STXeq/kg) for PSP toxins in seafood has been set on the basis of the MBA. However, MBA is not sensitive enough for trace analysis of PSP toxins in drinking water and it is not directly applicable to other matrices like micro algae. While different technologies for STX detection and analysis have been established, only a few Association of Official Analytical Chemists (AOAC) official methods are accepted; the mouse bioassay method [7], the pre-column oxidation [8,9], post-column oxidation HPLC-FLD method [10], and receptor binding assay (RBA) [11]. Human clinical samples are rarely available for testing of PSP intoxications, but, recently, a pre-column HPLC-FLD method was validated for the analysis of PSP toxins in clinical samples [12].

Recent interlaboratory validation studies were carried out mainly on mussel samples employing the official AOAC methods [13,14,15,16,17]. Based on the discussions in the EQuATox kick-off meeting in Berlin in March 2012, the main focus of STX PT was the identification and quantification of STX in real samples instead of spiked reference standards. The PT sample material was produced from naturally contaminated algae or shellfish. We present herein the results of the preparation and characterization of the PT sample material, homogeneity and stability studies of the PT samples, and reported PT results of the participating laboratories. In addition to the official AOAC methods, valuable information on the applicability of enzyme-linked immunoassay (ELISA), lateral flow assay, and liquid chromatography-tandem mass spectrometry (LC-MS/MS) methods for the analysis of PSP toxins was obtained from the participating laboratories.

## 2. Results and Discussion

### 2.1. Preparation and Characterization of STX PT Sample Material

Toxic algal cell sample material was obtained from *A. ostenfeldii* cultures grown at the Finnish Environment Institute (SYKE, Helsinki, Finland). This dinoflagellate produces STX, gonyautoxin 2 (GTX2) and gonyautoxin 3 (GTX3). Toxic mussel material was provided by the Marine Institute (Galway, Ireland). The toxin profile of the mussel material contained a mixture of STX and some main PSP toxin analogues *i.e.*, decarbamoyl saxitoxin (dcSTX), neosaxitoxin (NEO) and gonyautoxins (GTX1–5). The algal samples, both solid and liquid, contained only STX, GTX2 and GTX3, which are naturally produced by *A. ostenfeldii* in the Baltic Sea. The PSP toxins in naturally contaminated mussel samples were selected so that, in addition to STX, the mussel samples contained various closely related PSP toxins, which were also commercially available as reference standards and which had different substituents, such as carbamoyl, *N*-sulfocarbamoyl, hydroxyl, and sulfate groups. The selected PSP analogues with different charge states are affected by factors like pH and are thus challenging in sample preparation and analysis. Some of the PSP analogues can also be chemically converted from one to another e.g., during sample preparation and this may cause change in toxicity. The concentration level of PSP toxins in PT samples was fixed to be high enough for all analytical techniques; an exception was the most diluted STX PT sample (E1) in which the concentration level (4.7 ng/mL STX, Table 2) was adjusted to be close to the detection limit of chromatographic methods.

Both sample materials were characterized by two or three laboratories by three methods; pre-column oxidation high performance chromatography with fluorescence detection (HPLC-FLD), liquid chromatography-tandem mass spectrometry (LC-MS/MS), and enzyme-linked immunoassay (ELISA). The PSP toxin profiles of the samples are presented in Table 1. The PSP analogues were compared against the certified PSP reference standards obtained from the National Research Council (NRC, Canada). The identification of PSP toxins was based on the Organisation for the Prohibition of Chemical Weapons (OPCW) retention time criteria of |∆rt| ≤ 0.2 min [18] and on the specific fragmentation pattern of MS spectra when compared to the certified reference standards [19]. The presence of GTX4 in the mussel sample could not be confirmed before the PT because of the non-separable epimeric pair of GTX1 and GTX4 in pre-column oxidation method and the low sensitivity of GTX4 in the LC-MS/MS measurements.

**Table 1:** Characterization of paralytic shellfish poisoning (PSP) toxins in saxitoxin (STX) proficiency test (PT) sample material.

Method	Algal Sample Material	Mussel Sample Material
	PSP Analogues Identified	PSP Analogues Identified
Ridascreen ELISA	PSP positive	PSP positive
LC-MS/MS	STX, GTX2, GTX3	STX, dcSTX, NEO, GTX1, GTX2, GTX3
pre-column HPLC-FLD	STX, GTX2&3	STX, dcSTX, NEO, GTX1&4, GTX2&3, GTX5

The samples were quantified with pre-column oxidation HPLC-FLD [8] and LC-MS/MS method validated for algal samples [20]. The assigned values for STX in the samples are presented in Table 2.

**Table 2:** The assigned values and standard deviations for STX analyzed before the PT.

STX PT Sample	Sample Type	STX	Method
A	algal cells on filter paper	572 ± 75 ng/mL	LC-MS/MS
E1	algal extract (1:50)	4.7 ± 0.6 ng/mL	LC-MS/MS
E2	algal extract (1:10)	28 ± 2 ng/mL	LC-MS/MS
M	mussel sample	126 ± 0.6 ng/g mussel	HPLC-FLD

STX was identified in algal and mussel samples with two LC-MS/MS instruments either in the product ion scan or in the multiple reaction monitoring (MRM) modes. The chromatographic separation of PSP toxins was performed with hydrophilic interaction liquid chromatography (HILIC). The identification was based on the retention time and mass fragmentation criteria [18,19]. The retention times of the analytes were compared to the retention times of the certified reference standards. The mussel matrix affected the retention times and, thereafter, the presence of STX was confirmed with the signal increase after the addition of STX reference standard to the mussel matrix. The relative product ion intensities of the monitored fragment ions of the two distinguished LC-MS/MS methods fulfilled the EU criteria for both the algal and the mussel samples (Table 3).

**Table 3:** Retention time (rt, min) and relative product ion intensities (q/Q, %) based identification of STX in algal and mussel samples either with product ion scan or multiple reaction monitoring (MRM) mode, liquid chromatography-tandem mass spectrometry (LC-MS/MS) at *m*/*z* 300.

**LC-MS/MS**	**STX Standard (20–80 ng/mL, *n* = 3 × 7)**	**STX Algal Sample *n* = 90**	**STX Mussel Sample *n* = 17**	**Tolerance *^d^***
**rt**	6.32 ± 0.06 min	6.31 ± 0.06 min	*^c^*	±0.2 min
**Product Ion Scan *^a^ m/z***	**q/Q (%)**	**q/Q (%)**	**q/Q (%)**	**q/Q (%)**
266/282	19 ± 1	19 ± 2	18 ± 3	13–25
204/282	21 ± 1	22 ± 2	19 ± 2	16–26
186/282	9 ± 1	9 ± 1	8 ± 2	4–14
**LC-MS/MS**	**STX Standard (20–80 ng/mL, *n* = 3 × 7)**	**STX Algal Sample *n* = 90**	**STX Mussel Sample *n* = 17**	**Tolerance *^d^***
**rt**	15.2 ± 0.1 min	15.1 ± 0.1 min	*^c^*	±0.2 min
**MRM *^b^ m/z***	**q/Q (%)**	**q/Q (%)**	**q/Q (%)**	**q/Q (%)**
138/204	81 ± 4	68 ± 4	71 ± 6	65–97
282/204	96 ± 5	89 ± 3	94 ± 7	77–115

*^a^* Finnigan LXQ; *^b^* AB Sciex 3200 QTrap; *^c^* STX confirmed with the reference standard addition; *^d^* retention time tolerance |∆rt| ≤ 0.2 min [18]; EU criteria: compare with the relative ion intensity of the standard, q/Q >50%: tolerance ± 20%; >20% to 50%: tolerance ± 25%; >10% to 20%: tolerance ± 30%; ≤10%: tolerance ± 50% [19].

### 2.2. Preparation of the PT Samples

The summary of the four prepared STX PT samples is given in Table 4. Algal cells of PSP producing dinoflagellate *A. ostenfeldii* were filtered using filter paper. These filters represented the solid algal sample type (A) in the STX PT. Some of the algal samples on filter paper (A) were further extracted (*n* = 15), and two samples of algal extracts were prepared by diluting the pooled extracts 1:50 (E1) and 1:10 (E2) in acidified water (4 mM ammonium formate, pH 3.5 adjusted with formic acid). The fourth PT sample was a homogenized mussel sample (M). The levels of the total toxicity of algal and mussel samples were chosen to be toxic enough to be analyzed by MBA. Two concentration levels of the algal extracts were selected so that the higher concentration level of the PSP toxins would be close to the detection limit of MBA and the lower concentration would be near the detection limits and quantitation limits of more sensitive methods, respectively. Thus, the capability of various methods to detect minor amounts of PSP toxins could be assessed. 

**Table 4:** STX PT samples.

STX PT Sample	Sample Type	*n*	Sample Amount
A	Toxic freeze-dried algal sample on filter paper	120	~350,000 cells
E1	Pooled toxic algal extract (diluted 1:50)	47	5.0 mL
E2	Pooled toxic algal extract (diluted 1:10)	28	5.0 mL
M	Toxic homogenized mussel sample	100	~5.3 g

#### 2.2.1. Homogeneity of the Samples

The homogeneity studies of algal samples were performed using the Finnigan LXQ linear ion trap LC-MS/MS instrument in product ion scan mode. Algal cells on filter paper (A, *n* = 15) were extracted with the LC-MS/MS eluent (4 mM ammonium formate–ACN 40:60, pH 3.5 adjusted with formic acid), and the volume was adjusted to 2.0 mL. Two LC-MS/MS samples were prepared from the extracts, and STX in the samples was measured three times (*n* = 15 × 6). Ten parallel algal extract samples (*n* = 10) were measured twice (E1 and E2). Statistical analyses did not reveal any outliers (Table 5). Ten mussel samples were extracted in parallel and analyzed for all PSP toxins with pre-column oxidation HPLC-FLD. They were found sufficiently homogeneous with a relative standard deviation (RSD) of 5% for STX.

**Table 5:** Homogeneity results of STX PT samples.

STX PT Sample	Sample Type	*n*	STX in the Sample	RSD (%)	Method
A	Algal cells on filter paper	15	654 ± 77 ng on filter	12	LC-MS/MS
E1	Algal extract (1:50)	10	4.7 ± 0.6 ng/mL	14	LC-MS/MS
E2	Algal extract (1:10)	10	28 ± 2 ng/mL	8	LC-MS/MS
M	Mussel sample	10	130.7 ± 6.2 ng/g mussel	5	HPLC-FLD

#### 2.2.2. Stability of the Samples

Fifteen algal samples (A, E1, and E2) were randomly selected for the stability study of STX in algal cell samples and algal extracts. Parallel sets of three samples were kept for four weeks at 4 °C and at room temperature and also for six weeks at −20 °C, 4 °C, and at room temperature. The four-week stability samples were transferred to the freezer (−20 °C), and kept there for an additional two weeks prior to the STX analyses together with the six-week stability samples in the same LC-MS/MS batch. The algal samples had good stability for PT.

The Marine Institute in Ireland carried out a 32-day reverse isochronous stability study at −20 °C, 4 °C, and 40 °C for mussel samples. The samples were stored at –80 °C after preparation of the samples and before the start of each stability test. Three aliquots (*n* = 3) were measured at each temperature after storage time of 32, 11, five and three days, respectively. Analyses of STX, GTX2&GTX3, dcSTX, GTX5, GTX1&GTX4 and NEO were carried out from these samples with pre-column oxidation HPLC-FLD method. All PSP analytes in mussel samples showed excellent stability at −20 °C and 4 °C for at least 32 days.

**Figure 1:**
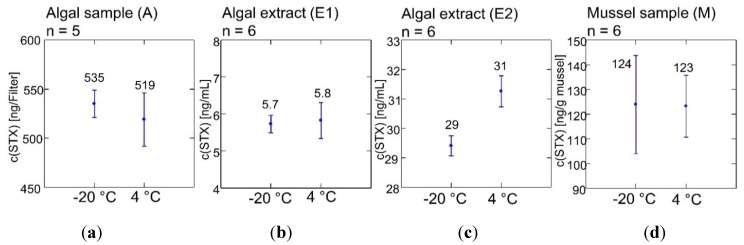
Four-week stability of the PT samples at 4 °C and −20 °C during the test. (**a**) Algal sample, A; (**b**) Algal extract, E1; (**c**) Algal extract, E2; (**d**) Mussel sample, M.

An additional stability study was performed during the PT. Statistically, all samples were found stable on the significance level of α = 0.05 when stored at 4 °C for four weeks (Figure 1). This was also the case when the calculated standard deviations (SD) for proficiency assessment were considered. If the criterion of ISO 13528 is taken as a basis, the highest σ_p_ (relative) was only 5.4% of the mean. One significant outlier was found in the algal sample batch (A), and this result was excluded (*n* = 5) from the calculations.

As a summary, all analytical data of the tested toxic algal samples and mussel samples indicate the suitability of the toxic freeze-dried algal material, the toxic algal extracts at two different dilutions and the toxic mussel sample as applicable sample matrices for this STX PT. The relative standard deviation for proficiency assessment, σ_p_ (relative), was set for each sample based on the homogeneity and stability data.

### 2.3. STX PT Results

Triplicate algal samples (A), triplicate mussel samples (M), and algal extracts at two toxin concentration levels (5.0 mL of both E1 and E2), altogether eight samples were sent to ten participating laboratories from eight countries. All STX PT samples were stored in a freezer for about two months at −20 °C by the test organizer, and analyzed once more just before the shipment. The samples were shipped to the laboratories under temperature-controlled conditions by express mail. Electronic reporting forms were provided via email (22 April 2013). The transportation reports indicated that the temperature of all sample packages has stayed during the whole transportation between 4.8 and 5.4 °C. All laboratories reported that the received sample packages were intact. The last package arrived at the participating laboratory in the morning of the third day after dispatch. The deadline for the reporting was within one month after the sample dispatch (22 May 2013). The main task for the laboratories was to report results for the presence of STX in all of the eight samples (A, E1–E2, M). All algal samples (A) and algal extracts (E1–E2) contained three different PSP toxins. The laboratories were asked to identify those toxins and additionally to quantify them. The mussel samples (M) contained eight different PSP toxins. The laboratories were asked to identify those toxins and additionally to quantify them. The quantitative analyses were asked to be carried out in triplicate, if possible. It was also noted that the sample E1 contained PSP toxins that were too low levels to be detected using MBA.

#### 2.3.1. PSP Toxin Results

All participating laboratories (*n* = 10) reported their results in time. Results of the PSP toxins in the samples are presented in Table 6. Four laboratories applied more than one method for the sample analyses. Reliable unambiguous identification of an analyte should be based on at least two independent analytical methods according to the OPCW. All analyzed samples were reported to be PSP positive by all participating laboratories.

**Table 6:** Summary of PSP toxin positive samples (+) of ten participating laboratories with any method.

STX PT Sample	Analyte	1	2	3	4	5	6	7	8	9	10
A	PSP toxin	+	na	+	+	+	+	+	+	+	na
E1	PSP toxin	+	na	+	+	+	+	+	+	+	na
E2	PSP toxin	+	na	+	+	+	+	+	+	+	na
M	PSP toxin	+	+	+	+	+	+	+	na	+	+

Note: na: not analyzed.

#### 2.3.2. Immunoassay Methods

Immunoassay methods were used by four laboratories. All four test samples were found PSP positive using the Ridascreen ELISA kit and the Abraxis kit (Table 7). The most diluted sample (E1, algal extract) was negative using a lateral flow assay (Jellett Rapid Test) as expected on the basis of the approximate detection limit of 300 µg STXeq/kg [21]. Immunoassay methods are sensitive for measuring PSP toxins at low levels, except for the Jellett test, which cannot be applied for the drinking water at health alert level. However, all used immunoassay methods cannot differentiate PSP congeners. The response of the various PSP toxins is dependent on the kit properties, and the cross-reactivity of PSP toxins can vary. Further, the results cannot be properly quantified. Immunoassay methods, especially lateral flow tests, have shown poor sensitivity towards *N*-hydroxylated toxins such as GTX1, GTX4 and NEO. These methods are specific for PSP toxins and good for screening, but they are not sufficient for the identification of saxitoxin. Moreover, it is likely that the total toxicity is underestimated because of the poor sensitivity of some PSP toxins.

**Table 7:** Immunoassay results of STX PT samples, PSP positive (+) or PSP negative (–).

Laboratory		4	5	5	6	7
	Method	Ridascreen	Ridascreen	Jellett	Jellett	Abraxis
Sample						
A		+	+	+	+	+
E1		+	+	−	−	+
E2		+	+	+	+	+
M		+	+	+	+	+

#### 2.3.3. Mouse Bioassay (MBA)

MBA was used only by two laboratories (Table 8). One participating laboratory used MBA for the analysis of mussel samples (M) and the other applied this method for the analysis of algal samples (A). Algal extracts (E1, E2) were not analyzed by MBA. MBA participants were not requested to analyze sample E1 due to toxin levels being below the limit of detection of the MBA method. So far, MBA is the only method to measure acute toxicity of samples, but this method provides no information on the toxin profiles present. The official MBA method is applicable only for mussel samples and it has not been validated for the algal matrix. MBA has also limited sensitivity and accuracy [22].

**Table 8:** Mouse bioassay (MBA) results of STX PT samples.

Laboratory		9	10
	Method	MBA	MBA
Sample			
A		+	na
E1		no	no
E2		na	na
M		na	+

Note: +: PSP positive; na: not analyzed; no: too dilute for MBA.

#### 2.3.4. Chromatographic Methods

Individual PSP analogues can be determined separately with chromatographic techniques; pre-column and post-column HPLC-FLD and LC-MS. STX was detected and identified with all chromatographic techniques applied in those samples that were analyzed (Table 9). However, limited information was received on the STX identification criteria used by the participating laboratories, and thus further evaluation of the qualitative results was not possible.

**Table 9:** Summary of qualitative STX results obtained with LC-MS/MS, pre-column HPLC-FLD (pre), and post-column HPLC-FLD (post) methods.

Laboratory		1	2	3	4	5	6	7	8	9	10
Method		LC-MS/MS	pre-ox	post-ox	LC-MS/MS	LC-MS/MS	pre-ox	-	LC-MS/MS	pre-ox	-
Sample	Analyte										
A	STX	+	na	+	+	+	+	na	+	+	na
E1	STX	+	na	+	+	+	+	na	+	+	na
E2	STX	+	na	+	+	+	+	na	+	+	na
M	STX	+	+	+	+	+	+	na	na	+	na

Note: +: STX positive; na: not analyzed.

Other PSP analogues were also screened with chromatographic techniques and the qualitative results are summarized in Table 10. Six laboratories reported GTX2&GTX3, either separately or together in mussel samples. Six laboratories had found dcSTX and five laboratories NEO in mussel samples. GTX1&GTX4 was reported by four laboratories, and GTX4 was actually reported only by one laboratory.

As a summary, only few laboratories were able to identify all PSP analogues in the STX PT samples. The main differences in the results are probably due to the variable sensitivity of the methods. In addition it is known that the pre-column method cannot differentiate between the epimeric pairs of GTX2&GTX3 and GTX1&GTX4. One laboratory (3) reported GTX4 in the mussel sample using the post-column oxidation HPLC-FLD method. GTX5 was reported only by two laboratories (six and nine). A reason for that may be the temporary unavailability of the certified GTX5 reference standard before and during the test.

**Table 10:** Summary of qualitative results of STX analogues found with LC-MS/MS, pre-column HPLC-FLD (pre-ox), and post-column HPLC-FLD (post-ox) in the samples (laboratories 1–10).

Laboratory		1	2	3	4	5	6	7	8	9	10
Method		-	pre-ox	post-ox	LC-MS/MS	LC-MS/MS	pre-ox	-	LC-MS/MS	pre-ox	-
Sample	Analyte										
A	GTX2				+	+					
	GTX3			+	+	+					
	GTX2&3						+		+	+	
E1	GTX2				+	+					
	GTX3			+		+					
	GTX2&3						+		+	+	
E2	GTX2				+	+					
	GTX3			+	+	+					
	GTX2&3						+		+	+	
M	dcSTX		+	+	+	+	+			+	
	GTX2		+	+	+	+					
	GTX3		+	+		+					
	GTX2&3						+			+	
	GTX1			+		+					
	GTX4			+							
	GTX1&4						+			+	
	NEO			+	+	+	+			+	
	GTX5						+			+	

#### 2.3.5. Quantitative Results

**Figure 2:**
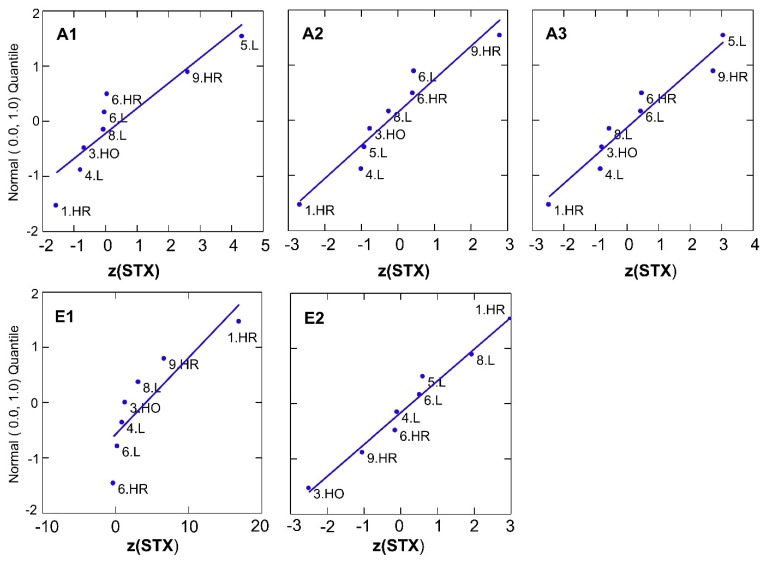

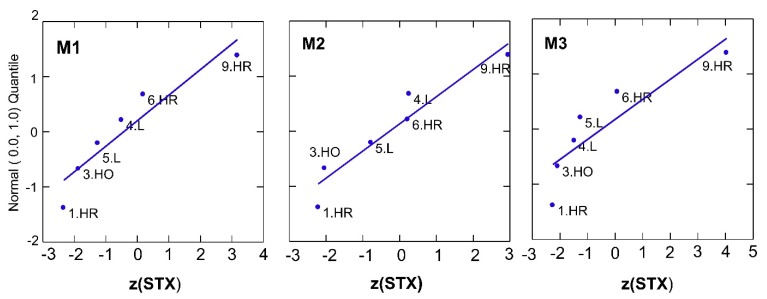
Graphical overview of the quantitative results of STX by normal probability plots of *z*-scores for A, E1–E2, and M. Participating laboratories 1–9, methods: LC-MS/MS (L), HPLC-FLD pre-column (HR) and post-column (HO).

The quantitative results on STX were statistically evaluated according to the recommendations of “The International Harmonized Protocol for the Proficiency Testing of Analytical Chemistry Laboratories” [23] and Algorithm A of the International Standard ISO 13528:2005 [24]. The quantitative results of STX are presented in Table 11 and Figure 2.

**Figure 3:**
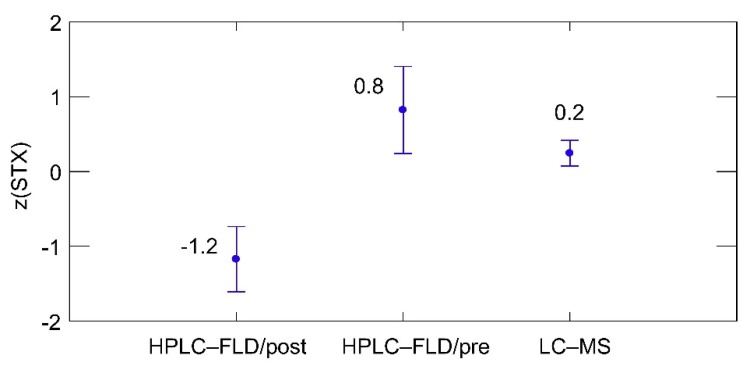
Effect of methods on the average quality of quantitation of STX, all samples, all methods, as assessed by the *z*-score means. Error bars represent ± 1 SD. Number of results: HPLC-FLD/post = 24, HPLD-FLD/pre = 56, LC-MS/MS = 75.

All chromatographic techniques applied for quantitative analysis of STX performed well with respect to trueness (Figure 3). The variation of quantitative results of STX was very small and mean of *z*-scores were within the range of −2 to +2. Moreover, quantitative LC-MS/MS results for STX were in good accordance with the official AOAC fluorescence methods. The agreement between laboratories using the same methods was also remarkable as indicated by the error bars.

**Figure 4:**
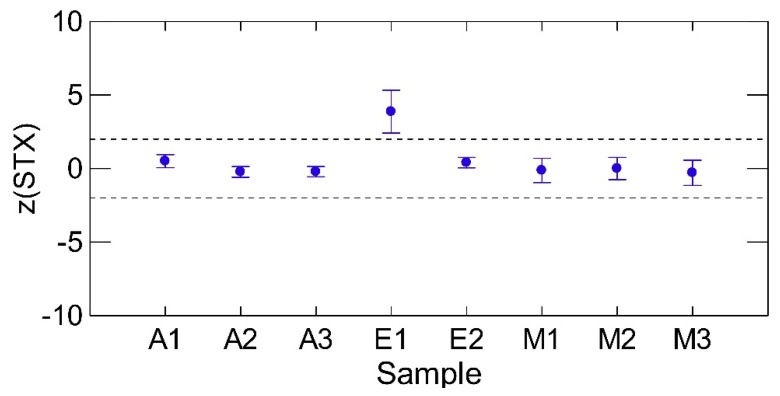
Effect of sample matrix on the average quality of quantitation of STX, all samples, all methods, as assessed by the *z*-score means. The dotted lines represent ±2 *z*-score values and the error bars represent ±1 standard error.

When the results of the different matrices were compared, a slightly larger variation in the mussel sample results was noticed and a significantly larger variation and deviation from the assigned value was detected in the most diluted sample E1 (Figure 4).

**Table 11:** Summary of reported STX concentrations, assigned values X_a_ (nominal), their standard and relative standard uncertainties and the standard deviations of the proficiency assessment.

Sample	µ	σ	Unit	*n*	CV	X_a_	u(X_a_)	u_rel_(X_a_) (%)	σ_p_
A1	621.7	276.4	ng STX/filter	8	0.445	572	19.4	3.4	145.9
A2	522.0	217.8	ng STX/filter	8	0.417	572	19.4	3.4	145.9
A3	609.6	309.4	ng STX/filter	8	0.508	572	19.4	3.4	145.9
E1	8.3	4.8	ng STX/mL	7	0.578	4.7	0.2	4.0	1.2
E2	30.0	13.7	ng STX/mL	8	0.455	28	0.6	2.3	7.1
M1	106.9	59.9	ng STX/g of mussel	6	0.560	126	0.3	0.3	32.1
M2	117.5	69.3	ng STX/g of mussel	6	0.590	126	0.3	0.3	32.1
M3	97.4	54.4	ng STX/g of mussel	6	0.559	126	0.3	0.3	32.1

Note: µ = mean of participants’ results as estimated by robust statistics; σ = standard deviation of participants’ means; *n* = number of mean results; CV = coefficient of variation (σ/µ); X_a_ = assigned value; u(X_a_) = standard uncertainty of the assigned value; u_rel_(X_a_) = relative standard uncertainty of the assigned value, u(X_a_)/X_a_ (%); σ_p_ = estimated standard deviation for proficiency assessment.

#### 2.3.6. Quantitative Results of other PSP Toxins

The concentrations of other PSP analogues were determined by two laboratories before the test with the pre-column oxidation HPLC-FLD method and are presented in Table 12. The total toxicity of the mussel sample had been set to about 1000 µg STXeq/kg, which was above the regulatory limit. The homogeneity and stability data for other PSP analogues was not thoroughly studied, and only few quantitative results were obtained for PSP analogues. Therefore, these results could not be properly statistically evaluated. The results for PSP toxins in mussel and algal samples are presented in Table 12, Table 13, Table 14 and Table 15.

**Table 12:** Concentrations of PSP toxins ng/g in mussel sample M.

ng/g Mussel	dcSTX	NEO	GTX1	GTX4	GTX1&4	GTX2	GTX3	GTX2&3	GTX5
**Before the PT**									
Ref. Lab 1 ^a^	169 ± 8	55 ± 3	na	na	316 ± 13	na	na	346 ± 24	2.9 ± 0.1
Ref. Lab 2 ^b^	194 ± 7	nq	na	na	430	na	na	515 ± 31	35
**PT results**									
*n*	15	15	6	3	6	6	6	6	6
mean	191	157	257	68	704	443	88	555	32
SD	66	154	118	3	409	161	15	40	5
CV%	35	98	46	4	58	36	17	7	16

Note: ^a^ Marine Institute; ^b^ SPIEZ LABORATORY; na: not analyzed; nq: not quantified; *n*: number of results.

**Table 13:** Gonyautoxin 2 (GTX2) and gonyautoxin 3 (GTX3) results in algal sample A (reference value ~4000 ng/filter for GTX2&GTX3 measured with HPLC-FLD).

PSP Analogue		GTX2	GTX3	GTX2&3
	***n***	6	9	6
**ng/filter**				
mean		1232	3803	3601
SD		415	850	1639
CV%		34	22	46

**Table 14:** GTX2 and GTX3 results in E1 (reference value ~40 ng/mL for GTX2&GTX3 measured with HPLC-FLD).

PSP Analogue		GTX2	GTX3	GTX2&3
	***n***	2	2	2
**ng/mL**				
mean		37	44	51
SD		13	25	3
CV%		37	58	6

**Table 15:** GTX2, and GTX3 results in E2 (reference value ~200 ng/mL for GTX2 & GTX3, measured with HPLC-FLD).

PSP Analogue		GTX2	GTX3	GTX2&3
	***n***	2	3	2
**ng/mL**				
mean		92	216	214
SD		6	75	121
CV%		6	35	57

## 3. Experimental Section

### 3.1. Material Preparation

#### 3.1.1. Toxic Freeze-Dried Algal Sample (STX PT Sample A)

Toxic algal samples were prepared at the Finnish Environment Institute. The PSP toxins producing strain AOF-0919 of *A. ostenfeldii* was cultivated at 16 °C under light-dark cycle of 14L:10D (*ca.* 60 µmol photons m^−2^s^−2^) in filtered, sterilized sea water (FSW) containing f/2-Si nutrients and having a salinity of 6 [25]. To grow the volume necessary for the analyses, 10 mL of an exponentially growing culture (AOF-0919) were inoculated into three replicate culture flasks containing 150 mL growth medium (FSW with f/2-Si nutrients) and grown for about one month. Subsequently, 7.4 mL of this inoculum culture were added to each of 30 sterile Nunc™ culture bottles (EasYFlask™ 75 cm^2^ with filter caps) filled with 150 mL of FSW with f/2-Si nutrients. The cell concentration of the inoculum was calculated to correspond to a final cell concentration of 1000 cells/mL in the experimental bottles. The cultivation was continued for about 5 weeks. The cell suspensions of the 30 culture bottles were combined, and the concentration of dinoflagellate cells was calculated (10,200 ± 700 cells/mL, *n* = 3) from this combined sample. Fractions of 35 mL from the combined cell suspensions were filtered on filter paper (GF/C, glass microfiber, Ø 25 mm, 1822-025, Whatman, Little Chalfont, UK), and washed with MilliQ water. The filter papers containing the dinoflagellate cells (~350,000 cells) were put in microcentrifuge tubes (conical, 2 mL, Molecular BioProducts, catalog #3468, Fisher Scientific, Loughborough, UK). The samples were freeze-dried, and the tubes sealed with caps (O-ring screw caps, Molecular BioProducts, catalog #3471Y, Fisher Scientific, Loughborough, UK). The produced samples (*n* = 120) were stored in a freezer at −20 °C.

#### 3.1.2. Toxic Algal Extracts (STX PT Samples E1 and E2)

Fifteen algal samples on filter paper were extracted with 2 × 1.0 mL of LC-MS/MS eluent (4 mM ammonium formate-ACN 40:60, pH 3.5 adjusted with formic acid), and the volume was adjusted to 2.0 mL. A pooled algal extract was prepared from the extracted algal samples (15 × 1.7 mL).

Preparation of the algal extract 1:50 (E1). 5.0 mL of pooled algal extract was diluted to 250.0 mL with 4 mM ammonium formate, pH 3.5 adjusted with formic acid (diluted algal extract, 1:50). 47 × 5.0 mL portions of the diluted algal extracts were measured in 8 mL glass vial (GF 61 × 16.6, 2411121634), capped with Teflon sealed caps, and stored in a freezer at −20 °C for PT samples.

Preparation of the algal extract 1:10 (E2). 15.0 mL of pooled algal extract was diluted to 150.0 mL with 4 mM ammonium formate, pH 3.5 adjusted with formic acid (diluted algal extract, 1:10). 28 × 5.0 mL portions of the diluted algal extracts were measured in 8 mL glass vial (GF 61 × 16.6, 2411121634), capped with Teflon sealed caps, and stored in a freezer at −20 °C for PT samples.

#### 3.1.3. Toxic Mussel Sample (STX PT Sample M)

Homogenized mussel samples (5 g, *n* = 100) were obtained from Marine Institute (Galway, Ireland) in October 2012. Toxic Spanish *Mytilus galloprovincialis* whole flesh tissue (500 g), toxic Canadian *Mytilus edulis* whole flesh tissue (7 g), and blank Irish *Mytilus edulis* mussel whole flesh tissue (893 g) were blended with stabilizers and antibiotics (0.02% of each ethoxyquin, ampicillin, erythromycin, and oxytetracycline) and the moisture content was adjusted to *ca.* 83.5% with additional water [26]. The mussel tissues were homogenized with Polytron^®^ 6100 (Kinematica™ AG, Luzern, Switzerland), and dispensed into 5 mL polypropylene tubes (Teklab Ltd., Sacriston, Durham, UK) with a peristaltic pump (Manostat, Barrington, IL, USA) adjusted to dispense *ca.* 5.3 g aliquots. The tubes were purged with nitrogen and hermetically sealed with aluminium seal closures (Seal-it-systems Ltd., Accrington, Lancashire, UK). Wadded screw caps were placed on the tubes. The frozen mussel samples were stored in a freezer at −20 °C.

### 3.2. ELISA Measurements of the Samples before PT

STX PT samples were analyzed with a competitive enzyme-linked immunoassay kit (Ridascreen Fast PSP SC, R1905, R-Biopharm, Darmstadt, Germany). The sample extracts were diluted in the ELISA buffer provided in the kit with two concentration levels so that the level of the PSP toxins would be between *ca.* 5–40 ng/mL. The sample analyses were performed with Ridascreen ELISA according to the kit instructions and specifications. The absorbances of the samples were measured in duplicate at 450 nm with a microplate spectrophotometer (Multiskan Go, Thermo Scientific, Vantaa, Finland). The zero standard (the PSP standard 1) was measured in duplicate as such from two separate wells (*n* = 4). The obtained average absorbance value of the zero standards was used for the calculation of corrected absorbances. The amount of the PSP toxins in the sample was calculated approximately with the RIDA^®^SOFT Win program with a cubic spline fitting of the standard curve.

### 3.3. Extraction of the Algal Samples for LC-MS/MS Analyses

The freeze-dried algal samples were extracted, centrifuged, and filtered. The algal sample on filter paper was transferred into 15 mL Falcon tubes (polypropylene, conical, 17 × 120 mm). The mass of the filter paper with algal material was measured, and the algal sample was extracted with extraction solvent (4 mM ammonium formate-ACN 40:60, pH 3.5 adjusted with formic acid). 1.0 mL of the extraction solvent was added into the Falcon tube, shaken for 2 min, allowed to stay in an ice bath for 15 min, and centrifuged with 3700 rpm (relative centrifugal force, RCF 2400× *g*) for 10 min at 4 °C. The sample was transferred into a centrifuge filter (PVDF Ultrafree, 0.45 µm, 0.5 mL, Millipore) and centrifuged with 14,000 rpm (RCF 21,500× *g*) for 5 min at 4 °C. The extraction was repeated with another 1.0 mL of extraction solvent, and the volume of the extract was adjusted to 2.0 mL with the extraction solvent.

### 3.4. Extraction of the Mussel Samples for LC-MS/MS

The mussel samples were extracted with the slightly modified AOAC 2011.02 method [10]. The mussel sample (5 g) was extracted with 4 mL of 1% acetic acid in MilliQ water by shaking the sample with Multi Reax mixer at room temperature for 30 min. The sample was heated at 95–100 °C for 5 min and mixed with Multi Reax mixer for 5 min at room temperature. The sample was cooled in an ice bath for 5 min and centrifuged with 5000 rpm (RCF 2400× *g*) for 10 min at 4 °C. The supernatant was decanted and the extraction was repeated with another 4 mL of 1% acetic acid in MilliQ water. The extraction solvents were combined and the volume was adjusted to 10.0 mL with 1% acetic acid in MilliQ water. The sample was transferred into a centrifuge filter (PVDF Ultrafree MC, 0.45 µm, 0.5 mL, Millipore, Carrigtwohill, Ireland) and centrifuged with 14,000 rpm (RCF 21,500× *g*) for 5 min at 4 °C. For the LC-MS/MS analyses, the samples were diluted 1:10 with LC-MS/MS eluent (4 mM ammonium formate-ACN 40:60, pH 3.5 adjusted with formic acid). The precipitate was filtered through a LCR filter (PTFE, 0.45 µm, Ø 13 mm, Millex, Merck Millipore, Carrigtwohill, Ireland) prior to the LC-MS/MS analyses. The detailed sample preparation and LC-MS/MS analyses of mussel extracts are described by Harju *et al.* [27].

### 3.5. LC-MS/MS Analyses

#### 3.5.1. LC-MS/MS Using the Product Ion Scan Mode for the Characterization, Homogeneity and Stability Studies of Algal and Mussel Samples before the PT

LC-MS/MS was performed using a Finnigan LXQ linear ion trap mass spectrometer with positive mode electrospray ionization (ESI) source interfaced to a Finnigan Surveyor Autosampler Plus Liquid Chromatograph (ThermoFinnigan, Hemel Hempstead, UK) with a method validated for algal samples [20]. The chromatographic separation was carried out on a TOSOH Bioscience HILIC TSK-gel Amide-80^®^ column (150 mm × 4.6 mm, 3 µm particle size, Stuttgart, Germany). A mobile phase was 4 mM ammonium formate-ACN 40:60, and the pH of the eluents was adjusted to 3.5 with formic acid. The flow rate was 1.0 mL/min with an accurate post-column splitter (1:20) between LC and MS. Spray voltage of 5 kV was applied and nitrogen was used as sheath gas. Capillary temperature was set to 350 °C and the relative collision energy was 29%. The quantitative analysis of STX in algal samples was performed with GTX1 (150 ng/mL) as an internal standard. The mussel samples were analyzed using only an external STX standard. The certified reference standards of PSP toxins were purchased from the National Research Council, NRC, Halifax, Canada (except GTX5, which was temporarily unavailable).

#### 3.5.2. LC-MS/MS with MRM for the Characterization of Algal and Mussel Samples before PT

The LC-MS/MS measurements were carried out on an Applied Biosystems 3200QTrap hybrid quadrupole-linear ion trap mass spectrometer (Applied Biosystems/MDS Sciex, Foster, CA, USA). The ion source was a “Turbo V”, SCIEX (Toronto, ON, Canada) which was operated in the positive ESI mode. The chromatographic system was an Agilent Series 1200 HPLC, Agilent Technologies AG (Basel, Switzerland). A Sequant ZIC-HILIC column 150 × 2.1 mm, 5 µm particle size (Merck, Darmstadt, Germany) in combination with a ZIC-HILIC guard column was used. The chromatographic conditions were chosen according to Turrell *et al.* [28]. A mobile phase consisting of two eluents was used: A (100% deionized water) and B (95%, *v/v*, acetonitrile). Both eluents contained 2 mM ammonium formate and 3.6 mM formic acid. Flow rate was 0.2 mL/min. The column was kept at 30 °C and the injection volume was 5 µL. The gradient was A: 50%–85% within 15 min, hold 5 min followed by an equilibration step at A: 50% for 5 min.

### 3.6. Pre-Column Oxidation HPLC-FLD Analyses

#### 3.6.1. HPLC-FLD Method Used for the Characterization of Sample Material before PT

The measurements were based on the procedures given in the AOAC Official Method 2005.06. An HPLC system from Thermo Fisher Scientific (Reinach, Switzerland) was used with the following components: UltiMate LPG-3400RS quaternary analytical pump, UltiMate 3000 analytical split-loop autosampler, UltiMate TCC-3000RS column thermostat and a fluorescence-detector RF-2000 with excitation wavelength at 340 nm and emission wavelength at 400 nm. The chromatographic conditions were chosen according to AOAC. A Supelcosil C18 column (150 × 4.6 mm, 5 µm particle size) from Sigma-Aldrich Chemie GmbH (Buchs, Switzerland) was used. The eluent consisted of two phases: A: 0.1 M ammonium formate (5% acetonitrile), B: 0.1 M ammonium formate. Flow rate was 2 mL/min. The gradient conditions were: initially 0% A, hold for 1 min, increase to 5% in 4 min, increase 5%–70% in 5 min, hold for 2 min and return to 0% A and re-equilibrate for 5 min. The column temperature was kept at 25 °C.

#### 3.6.2. HPLC-FLD Method Used for the Homogeneity and Stability Studies of Mussel Samples before PT

Samples were extracted, oxidized and analyzed closely following OMA AOAC 2005.06. A Shimadzu (Kyoto, Japan) LC system with a fluorescence (FLD) detector (ex 340 nm, em 395 nm) (Shimadzu RF-10AXL) and cooled autosampler (Shimadzu SIL-20A) was used. The LC column was a reverse phase C-18 Supelcosil (150 mm × 4.6 mm, 5 µm) fitted with a C-18 Supelguard cartridge (20 mm). The LC programme followed was a slightly modified gradient elution based on that published in AOAC 2005.06 using a flow rate of 1.5 mL/min. The gradient followed was 0%–5% mobile phase B over 5 min, 5%–70% B over the next 4 min, back to 0% B over 2 min, then keeping at this condition for 7 min before the next injection. Toxin concentrations in sample extracts were quantified against a five-point calibration. All homogeneity and stability study extracts were analyzed in the same chromatographic sequence to negate day-to-day instrument variations.

### 3.7. MBA Tests

National authority permissions for the use of MBA were asked from those laboratories, which used MBA method in the PT.

## 4. Conclusions

We have shown the suitability of the algal and mussel sample material for the PT. The prepared reference material was characterized by various methods and the homogeneity and the stability of the STX in the samples were assessed prior to the test. Data obtained from the participating laboratories showed that PSP toxins could be detected in all samples, but MBA and immunoassay methods cannot differentiate PSP congeners. When compared to MBA, immunoassay methods are specific for PSP toxins but cannot measure acute toxicity of the samples. MBA turned out not to be applicable to matrices other than mussel tissue. Most participating laboratories performed well with respect to the identification and quantification of STX, though quantitative STX results on the most diluted algal extract varied and the coefficients of variation were quite high (42%–59%) for all samples. Limited information was obtained for other PSP toxins, and more work on the quantification of STX analogues is needed in the view of the fact that the chemical analyses of STX analogues will replace the MBA in the future. The quantitative LC-MS/MS results for STX were in good agreement with the fluorescence results measured with HPLC-FLD.
